# Cyclodextrins: Emerging Medicines of the New Millennium

**DOI:** 10.3390/biom9120801

**Published:** 2019-11-28

**Authors:** Susana Santos Braga

**Affiliations:** QOPNA & LAQV/REQUIMTE. Chemistry Department, University of Aveiro, 3810-193 Aveiro, Portugal; sbraga@ua.pt; Tel.: +351-234370200

**Keywords:** cyclodextrin, cholesterol, infection, intrathecal, acetylcholine, biomaterials

## Abstract

Cyclodextrins, since their discovery in the late 19th century, were mainly regarded as excipients. Nevertheless, developments in cyclodextrin research have shown that some of these hosts can capture and include biomolecules, highlighting fatty acids and cholesterol, which implies that they are not inert and that their action may be used in specific medicinal purposes. The present review, centered on literature reports from the year 2000 until the present day, presents a comprehensive description of the known biological activities of cyclodextrins and their implications for medicinal applications. The paper is divided into two main sections, one devoted to the properties and applications of cyclodextrins as active pharmaceutical ingredients in a variety of pathologies, from infectious ailments to cardiovascular dysfunctions and metabolic diseases. The second section is dedicated to the use of cyclodextrins in a range of biomedical technologies.

## 1. Introduction

### 1.1. Historical Overview

**Figure 1:**
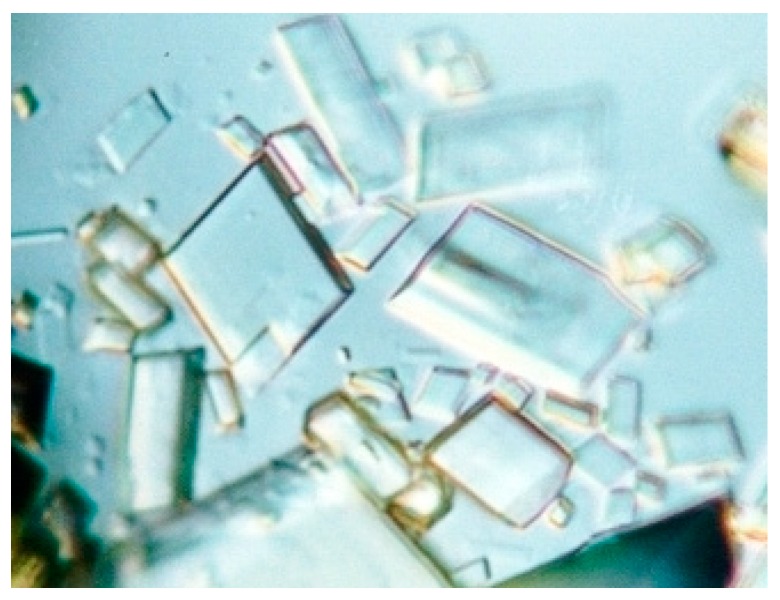
Crystals of a β-cyclodextrin complex with a small-sized guest (*p*-hydroxybenzaldehyde [2]), which illustrate the brightness of cyclodextrin crystals, first described by Villiers [1].

Cyclodextrins (CDs), first reported by Villiers in 1891 [1], have completed their 128th anniversary. Described by Villiers as carbohydrates that precipitate slowly (amidst the fermentation products of starch) in the form of “beautiful radiant crystals” (Figure 1), these molecules have kept as much attractiveness as mystery through the first five decades that followed their discovery.

In the early 50 years of their knowledge to humans, cyclodextrins were the subject of intense scientific curiosity [3]. Scientists of different groups sought to understand the origins of their formation, their reactivity (including the ability to host other molecules) and, most importantly, their structure. The cyclic nature of cyclodextrins was postulated in 1939 [4]; however, this first proposal, based on modelling alone, has also erroneously defined α-CD—the smallest cyclodextrin—as having five glucose units [4,5]. It was not until 1948, with the adequate purification and crystal structure resolution of each of the native cyclodextrins, that their composition was accurately defined, being of six, seven, and eight glucose units for α-CD, β-CD, and γ-CD [6].

In the second half of the 20th century, as the structure and properties of cyclodextrins became known with greater detail, studies were directed towards the exploration of their ability to form inclusion complexes with various molecules [7]. Cyclodextrins were found to protect sensitive organic guest molecules from volatilization and from oxidation and their solubilizing action on apolar guests made them attractive for a variety of applications [8]. When the industrial production of cyclodextrins started to make them available in larger quantities and the toxicological safety was ascertained (details in the Section 1.2.), applications in the pharmaceutical, cosmetic, and food chemistry have blossomed, and, more recently, applications expanded to (re-)emerging areas as nutraceutics and natural products [9,10,11]. In all of these products, cyclodextrins were essentially regarded as excipients or inert materials.

With the turn of the new millennium, developments in cyclodextrin biomedical research have once more surprised the scientific community by demonstrating that these molecules are not quite so inert and they that may, in fact, be used to treat some human ailments. Such medicinal properties are the main topic of this review and are presented in detail in Section 2.

### 1.2. Regulatory Status of Cyclodextrins

Native CDs are regarded in Japan as natural products, and for this reason they are used without many restrictions both in medicines and in foods. In western countries, the ingestion of native cyclodextrins is regulated by the JECFA (Joint WHO/FAO Expert Committee on Food Additives) [12,13,14], with the pharmaceutical applications falling under the European Medicines Agency (EMA) in Europe and under the Food and Drug Administration in the United States of America. Native CDs can be ingested without significant absorption, being thus ‘Generally Regarded As Safe’ by the FDA [15,16,17]; they are commonly referred to as molecules with ‘GRAS status’. α-CD and γ-CD can be taken without restrictions [12,14] while the oral intake of β-CD should be limited to a maximum of 5 mg per kilogram of weight each day [13]. Regarding parenteral use, native cyclodextrins suffer from much stronger restrictions. Indeed, the EMA recommends against the administration of α-CD and β-CD directly into the bloodstream due to renal toxicity [18]. In addition, native CDs are known to cause hemolysis in vitro, at concentrations of 6, 3, and 16 mM for α-, β-, and γ-CDs, respectively [19], due to extraction of phospholipids and cholesterol from the erythrocyte membrane [20].

Native CDs can be functionalized to afford a large variety and number of derivatives, surpassing 1500 different molecules according to a report of 2012 [21]. Of these, only a few are approved for human use in the fields of pharmaceutics. The U.S. Food and Drug Administration (FDA) lists 2-hydroxypropyl-β-cyclodextrin (HPβCD) and 2-hydroxypropyl-γ-CD (HPγCD) as approved inert materials (excipients), with HPβCD being suited for oral and intravenous administration while HPγCD can only be used in topical products and in a maximal concentration of 1.5% (*w*/*v*) (FDA, 2016). Within *O*-methylated CDs, the approval status varies from one molecule to the next. For instance, heptakis-2,3,6-tris-*O*-methyl β-CD (TRIMEB) is deemed unsafe for human use due to its hemolytic action and renal toxicity. Its sister cyclodextrin, heptakis-2,6-di-*O*-methyl-β-CD (DIMEB), also features some toxicity, mostly targeting the liver: Doses of 300 mg/kg in mice caused elevated levels of glutamate-pyruvate transaminase (GPT) and glutamate-oxaloacetate transaminase (GOT) [22], two biomarkers of hepatic injury. Despite this, DIMEB is approved by the FDA for commercial use in a few injectable vaccines [23], probably due to the fact that it is present in low amounts in such products. Cyclodextrins that have undergone *O*-methylation in random positions have different safety profiles, according to the different degrees of substitution. RAMEB (from randomly methylated beta-cyclodextrin), with an average of 1.8 methoxyl groups per glucose unit, has some hydrolytical action on erythrocytes [24,25], as well as renal toxicity that is higher than that of the parent βCD. For these reasons, RAMEB is not recommended for parenteral use by the EMA [15]. CRYSMEB (named after the fact that it is a crystalline solid, crystalline methylated beta-cyclodextrin) has a deliberately low substitution degree (average of 0.56 methyl groups per glucose unit, i.e., only four methyl groups in each CD molecule) because it was designed for high biotolerability. CRYSMEB does not cause hemolysis and it is already approved for dermal applications and as an ingredient in cosmetics. Another biocompatible CD is sulfobutyl ether β-CD (SBEβCD), developed to be non-nephrotoxic and present in several FDA-approved marketed medications for both oral and intravenous administration [26].

## 2. Medicinal Cyclodextrins

### 2.1. The Early Steps

Cyclodextrins were, for many years, regarded as excipients with solubilizing and stabilizing properties. Only in the end of the 20th century did scientists develop the concept of employing cyclodextrins as medicinal compounds. This discovery was part of the research for new polysulfated-based drug candidates to treat HIV (human immunodeficiency virus), which used cyclodextrins as scaffolds for sulfonation. Native cyclodextrins, with a large number of hydroxyl groups available, allowed the preparation of dozens of sulfonated derivatives by varying the substituents and degrees of substitution [27]. Sulfonated CDs were shown to reduce the infectivity of HIV and stop viral replication in vitro [27,28,29]. For tetradecasulfate-β-CD, the mechanisms of activity were shown to involve inhibition of the enzyme reverse transcriptase and inhibition of the ability of HIV to fuse with target cells [28]. These cyclodextrins were, however, never marketed for HIV therapy, which may be in part associated with reports of resistance to sulfonated CDs by HIV [30].

In the first years of the 2000s, identification of cholesterol as a target for HIV therapy [31] has rekindled the interest in the medicinal use of cyclodextrins, not only for HIV, as detailed in the following subsection, but in a variety of infectious diseases with pathogens that depend on membrane cholesterol for internalization into the host cells. Many cyclodextrins are able to bind to cholesterol, but those with a higher sequestering ability are β-CD and RAMEB, followed by DIMEB and HPβCD and then TRIMEB, as determined in vitro in human vascular endothelial cells [32]. The high biocompatibility of HPβCD makes it, typically, the cyclodextrin of choice for medicinal applications that require cholesterol sequestering.

### 2.2. Antiviral Activity

#### 2.2.1. HIV Management

HPβCD is able to remove some cholesterol molecules of the membrane of host cells, rendering them less susceptible to viral infection [31]. It also removes cholesterol of viral particles, and by this mechanism it causes disruption of both HIV and SIV (simian immunodeficiency virus) [33,34]. The ability to block the transmission of the virus was demonstrated by studies in mice, with vaginal application of HPβCD blocking HIV transmission by 91% (10 out of the 11 mice treated with HPβCD did not get infected following HIV exposure) [35]. Furthermore, HPβCD was shown to reduce the inflammatory response of cultured immune cells, lowering their production of interleukins (IL-10) and cytokines (TNF-α) [36].

The encouraging results, both in vitro and with mice, triggered several research projects aimed at obtaining a vaccine for HIV based on HPβCD. The next obvious step was to study the effectiveness in an animal model closer to humans, such as the rhesus macaque. The animals were treated with HPβCD by intravaginal administration and then subjected to SIV contact, with HPβCD protecting them from infection upon first contact with the virus. However, repeating the viral inoculation 11 or 47 weeks later, also under treatment with HPβCD, led to large-scale infection [37]. This means that HPβCD, at the tested doses, is unsuited as a vaccine for repeated exposure to the virus. Further studies are still needed to validate this medicinal application for HPβCD.

#### 2.2.2. Influenza Treatment and Prevention

**Figure 2:**
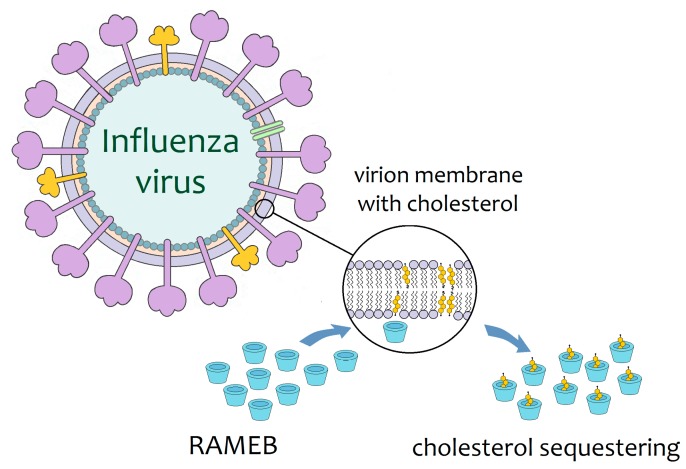
Schematic representation of the mode of action proposed for the RAMEB against influenza viral particles. RAMEB sequesters membrane cholesterol, resulting in damage to the integrity of the viral envelope [38].

The ability of cyclodextrins to sequester cholesterol allows them to potentially disrupt any virus with this biomolecule in its membrane. Membrane cholesterol is usually located in lipidic microdomains that are called lipid rafts. Particles of the influenza A virus, when treated with RAMEB for cholesterol depletion (Figure 2), exhibit disruption of lipid rafts, with consequent structural deformations of the viral membrane; microscopy examination allows observation of holes in the viral envelope [38]. Furthermore, cholesterol depletion by RAMEB significantly reduces the infectivity of viral particles of influenza A (H1N1 strain) in vitro [39,40]. Although RAMEB is not yet regulated for human use, these preliminary results have opened the way for the application of other cyclodextrins in the control of influenza infection in humans.

New cyclodextrin derivatives designed for the treatment of influenza include a family of fullerene-cyclodextrin conjugates [41] and another family of pentacyclic triterpene-functionalized per-(2,3-di-*O*-methyl)-α-, β-, and γ-CD derivatives [42,43]. The terpenic β-CD derivatives were the most potent, with IC_50_ values as low as 4.7 μM against virus cultivated inside canine epithelial kidney cells (MDCK line); their mechanism of action is to bind and block hemagglutinin, a protein on the virus surface, thus preventing its interaction with the host receptor and the entry of the virus into the host cells [42]. The new CDs are non-toxic against host cells and they seem to be promising new molecules for antiviral action, but the data is still limited to in vitro studies, and more research needs to be conducted on these molecules to fully understand their practical utility.

The antiviral properties of cyclodextrins also served as a base for the development of an innovative influenza vaccine by a research group from Japan. The vaccine is innovative as it uses the nasal delivery route to afford a patient-friendly administration, expected to be better accepted than the currently available injections. Another innovative aspect of the vaccine lies in the presence of HPβCD as an adjuvant agent for active immunoglobulins. HPβCD, already approved as an excipient for various oral medicines and a few injectable ones, was chosen for its excellent tolerability. The synergic effect of HPβCD with immunoglobulins was proven by several previous in vivo studies with mice and macaques, which demonstrated that HPβCD brings a 30% increase in the production of antibodies and that it further induces the production of a subclass of immune cells responsible for long-term immune ‘memory’; that is, long-term immunization against the virus [44,45]. It was also demonstrated that the effect of immunization occurs not only at the nasal mucosa, the site of administration, but that it is systemic, spreading throughout the entire body to convey strong immunity to the virus [46]. The vaccine is undergoing phase I clinical studies, which started in October 2017 and continue until the present day [47]. If successful, it will be the first cyclodextrin-adjuvanted vaccine in history.

#### 2.2.3. Interactions with the Dengue Virus

The depletion of cholesterol by RAMEB in virus from the Flaviviridae family was shown to affect flaviviral entry into the target cells as well as their ability to replicate inside the cells [48]. This was considered a very promising result for the management of infections from this family, which includes dengue virus (DEN) and Japanese encephalitis virus (JEV).

Dengue fever, with a high incidence in tropical and sub-tropical countries, was the object of a several studies concerning potential therapeutic applications of cyclodextrins. In vitro assays with a monocyte cell model (U937 myelomonocyte cell line) confirmed a significant reduction in infection rates upon treatment with RAMEB (30 mM) [49], which is associated with the inability of the viral particles to release their genetic material into the infected cells [50].

More importantly, RAMEB can interfere with the virus life cycle when it is inside the carriers, the Asian tiger mosquito (*Aedes albopictus*) and the mosquito (*Aedes aegypti*). RAMEB alters the protein metabolism of the virus inside the mosquito cells, namely by lowering the expression of NS1 (non-structural protein 1) [51]. Considering that NS1 is a protein required for viral replication and excretion, a reduction of viral secretion in the cultivated mosquito cells treated with RAMEB was expected; however, it did not occur. Further investigation on the possible application of RAMEB in the prevention of dengue transmission is needed, especially by studying the effect on mosquito larvae. If successful, this new methodology would bring an innovative and eco-friendly method for the environmental control of dengue.

#### 2.2.4. Other Pathogenic Viruses Targeted by CDs

The high cholesterol-binding properties of HPβCD and RAMEB led to research investigating their potential to act as medicinal molecules against a variety of viral agents for common infections. Any viral particle that has cholesterol in the exogenous part of its particles can be the target of these CDs. The ability of these CDs to reduce viral infectivity has already been demonstrated against several common viruses, like human metapneumovirus (HMPV) [52], parainfluenza virus type 3 (HPIV3) [53], coronavirus infectious bronchitis virus (IBV) [54], herpes simplex virus 1 (HSV-1) [55,56], and even a less common one, the Newcastle disease virus (NDV) [57,58].

HPβCD and/or RAMEB also interfere with the infectivity of viruses responsible for chronic infections, namely the varicela-zooster virus (VZV) that causes chickenpox [59], and the hepatitis C virus (HCV) [60,61]. For the treatment of hepatitis C, new cyclodextrins are being developed based on fullerene conjugation (in a similar way to some CDs described for influenza treatment). A conjugate of fullerene (C_60_) with two α-CD units was designed to combine the antiviral properties of C_60_ with good aqueous solubility. This molecule presents an IC_50_ of 0.17 μM against HCV and it is described as the first of a ‘new class of HCV entry inhibitor’ [62].

### 2.3. Antiparasitic Activity

#### 2.3.1. Leishmanicidal Cyclodextrins

Leishmaniasis is a neglected tropical disease endemic to tropical countries of Central and South Americas, Africa, and the Middle East, and is threatening to spread to the USA and south Europe due to global warming [63]. HPβCD, combining a good cholesterol-sequestering ability with approval for use in injectable formulations, is an excellent candidate for leishmaniasis therapy. In BALB/c mice infected with the parasite *Leishmania donovani*, administration of an aqueous solution of HPβCD (c.a. 320 μM, which corresponds to 448 mg/L) caused a 21% reduction of liver infection when compared to the control [64].

RAMEB has also been proposed as a new leishmanicidal drug, with studies demonstrating its effectiveness in reducing the infectivity of *L. donovani* and its ability to infect the immune cells of the host [65,66]. There is also a patent on the leishmanicidal use of RAMEB, in doses of 20 to 500 mg/kg of body weight and comprising a variety of administration routes, including oral, inhalable, and implantable [67]. Nevertheless, this product is, most likely, still far from reaching the market since the toxicity of RAMEB has not been fully elucidated and this cyclodextrin is not regulated for oral administration [18] by agencies, such as the EMA and FDA.

#### 2.3.2. Sulphated Cyclodextrins against Malaria

Malaria, a tropical hemorrhagic fever caused by protozoa parasites of the *Plasmodium* genus, is a clinically challenging disease due to the increase of strains resistant against the most commonly used medication, chloroquine. The search for new medications has demonstrated that anionic saccharides are effective in blocking the parasite from entering target cells, specifically erythrocytes [68] and hepatocytes. Based on these findings, anionic cyclodextrins, namely with sulphate substituents, were prepared and tested as antimalarial agents on cultured *Plasmodium falciparum* [69]. Results showed that the size of the cyclodextrin ring is not a critical factor for the activity, as derivatives from all the parent cyclodextrins (α-, β-, and γ-CDs) inhibited parasite replication. The potency of the activity of each cyclodextrin derivative against the malaria parasites seems to mainly be related to the degree of substitution. Indeed, the most potent compound was the sodium salt of a poly-sulphated β-cyclodextrin with 16.9 sulphate groups (per CD molecule), the highest average degree of sulphation tested. This derivative exhibited an IC_50_ value of 2.4 ± 0.3 μM against *P. falciparum*. Also noteworthy is the fact that CD derivatives with a low degree of sulphation (0.8 to 1.7) were completely inactive. These results show that 16.9-sulphated-β-CD could be a promising new lead for malaria treatment, although a thorough investigation of the safety profile of this molecule is still needed for it to make a successful transition into clinical applications.

#### 2.3.3. Cyclodextrins against Cryptosporidiosis

Cryptosporidiosis is an enteric infection caused by parasites of the species *Cryptosporirium parvum* and it typically manifests as watery diarrhea. The strong loss of liquids is of greater concern in babies, infants, and pregnant women. In immunocompromised patients, cryptosporidiosis is a severe infection and it often leads to the death of HIV co-infected patients. The disease is transmitted by water contaminated with oocysts of *C. parvum*, each one releasing four infectious sporozoites once inside the intestines of the host. Research conducted by a team from Galicia, Spain, in the beginning of the 21st century, demonstrated that two native cyclodextrins, α-CD and β-CD, are very useful for the control of criptosporidiosis as they reduce the viability of the oocysts [70]. Treating contaminated water with β-CD (25 g/L) for 24 h allowed a reduction of the load of infection in mice by 77%, when compared to mice exposed to the same amount of contaminated water that was not treated with β-CD; administering an aqueous suspension of β-CD to infected mice also showed curative properties, with treatment rates being most effective when β-CD was given only 8 h after infection [71].

Cryptosporidiosis also has a strong impact in animal farming, because the main reservoirs of *C. parvum* are animals of the bovine, caprine, and ovine species. Cryptosporidiosis causes significant economic losses in the breeding and farming of these animals and there is a strong demand for new therapeutics. With this in mind, β-CD was evaluated against lamb cryptosporidiosis under field conditions. A single daily dose of 500 mg/kg of body weight, administered for three consecutive days, was shown to reduce both the clinical symptoms and the intensity of infection in the lambs. Furthermore, prophylactic administration of β-CD within 24 h of birth of the newborn lambs reduced the mortality rate and the number of infected newborns [72].

Veterinary application of α-CD was studied in newborn goats, artificially inoculated with *C. parvum* in laboratory-controlled conditions. Results showed that a daily dose of α-CD at 500 mg/kg of body weight (distributed over four intakes) during six consecutive days was able to prevent diarrhea onset in 83% of the cases (five of six goats) and could reduce the parasitic load in the faces of these animals [73]. Considering that α-CD is prone to interaction with various components of the milk that serves as nourishment for these goat kids, a higher dosage and perhaps a more prolonged treatment time would be worth investigating to determine the optimal therapeutic plan. Further studies could also include clinical trials on humans, because α-CD is quite safe, it has no dietary intake limits, and it is already used as a nutraceutical in beverages [74]. In a scenario of cryptosporidiosis-caused diarrhea, α-CD-containing beverages would be most adequate as they would help ameliorate both dehydration and infection.

### 2.4. Cyclodextrins in Cardiovascular Diseases

Atherosclerosis, the root cause of heart attack, stroke, and peripheral vascular disease, is a pathology of the arterial wall. It develops progressively by the accumulation of cholesterol-rich lipids on sensitive spots of the arteries to form plaques. In these plaques, oxidized cholesterol is thought to form a blockage that prevents the normal mechanisms of the organism from removing the lipid build-up [75]. The discovery that HPβCD can solubilize these forms of cholesterol provided a new hope for reverting the accumulation of atherosclerosis plaque [76]. In vivo studies with mice showed that HPβCD is well tolerated at daily doses of 13 mg/day over four weeks, but it affords no reduction in total cholesterol plasma levels [77]. Another set of in vivo studies with mice has shown that, although plasma cholesterol is not altered, HPβCD fights atherosclerosis by helping dissolve cholesterol deposits in the arterial walls and reprogramming macrophages to metabolize cholesterol into soluble oxysterols [78]. On excised human atherosclerotic carotid plaques, HPβCD exhibits anti-inflammatory action by regulating complement-related genes in the cells of the plaque and reducing the levels of a pro-inflammatory molecule, complement component C5 [79].

The role of α-CD in cardiovascular disease prevention was also investigated. Given that α-CD is already approved as a nutritional supplement, the study, conducted on a group of 75 healthy volunteers, focused on oral administration of a daily dose of 6 mg for 12 weeks. No significant beneficial changes in the profile of plasma lipids were, however, observed [80]. This can be attributed to the quasi null absorption of α-CD from oral intake. Note also that the effects of the cyclodextrin in blocking the absorption of dietary lipids were not measured in this study.

### 2.5. HPβCD under Clinical Trials for Focal Segmental Glomerulosclerosis

The well-known cholesterol-binding ability of HPβCD serves as base for its mode of action against this rare kidney disease. In focal segmental glomerulosclerosis (FSGS), cholesterol and lipids accumulate in the glomeruli (the kidney cells responsible for filtration), which become damaged and leak proteins into the urine. The disease is often associated with hypoalbuminemia and hypertension. There is no available medication and patients frequently require renal transplant. HPβCD, by removing the excess cholesterol from the kidneys, restores renal function, albumin levels, and the body’s homeostasis [81]. The use of HPβCD for the treatment of FSGS has successfully completed phase I clinical studies (in mice), and it was approved by the FDA in April 2018 for phase IIa trials on human patients [82].

### 2.6. HPβCD, an Orphan Drug for Niemann–Pick Disease Type C

Niemann–Pick disease (NPD) of type C is a genetic neurodegenerative disorder characterized by the abnormal accumulation of cholesterol and sphingolipids inside the lysosomes of the affected cells due to failure in the gene that encodes the transport protein NPC1, responsible for the mobilization of these water-insoluble biomolecules (note also that this feature distinguishes NPD type C from the types A and B, which are associated with genes encoding sphingomyelinase). It affects brain cells quite strongly, triggering severe neurological symptoms, such as ataxia, tremors, loss of muscle tone, and loss of vision. The liver and spleen may also be affected, typically displaying enlargement. NPD has a higher incidence in children, and it causes progressive loss of bodily control and function, ultimately leading to death [83].

The lack of medication against NPC encouraged the search for innovative medication to remove lipid deposits in the brains of these patients. HPβCD is an obvious choice for its cholesterol-sequestering properties. Preliminary studies were conducted in a mouse model by intravenous administration. As no results were observed, it was concluded that HPβCD is unable to cross the blood–brain barrier [84] and that in order for HPβCD to be able to treat NPD, it needs to be administered in a more invasive way, that is, directly into the spinal canal (intrathecal administration). Administration of HPβCD by the intrathecal route showed positive results in mouse [85] and cat [86] models of the disease, with a delay in the progression of neurological damage and partial recovery of swelling in a few brain regions.

HPβCD was approved as an orphan drug for NPD type C by the FDA in 2010 [87] and by the EMA in 2013 [88]. Since then, it was used intrathecally in various clinical trials, some of which have already ended. One of these trials showed restoration of neuronal cholesterol homoeostasis and a decrease in CNS pathology in children less than 18 months of age [89]. In three children aged from 30 to 36 months, HPβCD improved scores on the cognitive level, swallowing ability, fine motor skills, and balance/gait [90]. Another trial with 14 participants of a wider scale of ages, from 4 to 23 years, confirmed that there is an overall slowing of the progression of the disease upon intrathecal administration of HPβCD (at doses of up to 600 to 1200 mg/month, according to the tolerance of each patient); the patients were medicated for three years [91]. Many other clinical trials are still ongoing or in the starting phase, with a total of nine currently active clinical trials with HPβCD [92] that engage a large number and variety of medical institutions around the globe. Hopefully, these will help elucidate the role of HPβCD, its long-term toxicological safety, and its biological fate. So far, knowledge is limited to proteomic analysis of the effect of HPβCD on cultured fibroblasts from donors with NPD type C, showing that HPβCD increases the production of LAMP-1 and other proteins involved in carrying lipids out of the cells [93].

### 2.7. Sugammadex, a New Drug for Quick Reversal of Neuromuscular Blockage

Neuromuscular blocking agents (also known as skeletal muscle relaxants) are molecules able to block the effects of acetylcholine by occupying its place at nicotinic receptors in striated muscle cells. They are commonly used during surgical procedures to relax the muscles of the patient, ensuring immobilization and facilitating the insertion of ventilation tubes in the trachea [94]. When the surgical procedure is finished, the removal of neuromuscular blockage is usually done by administering a reversal agent, to make sure the patient regains control of his muscular function as quickly as possible and that artificial ventilation can be removed. These agents are usually acetylcholinesterase inhibitors, such as neostignime, and they lead to a strong increase in acetylcholine levels. The high concentration of acetylcholine drives it to replace the blocking agent at the nicotinic receptors, restoring normal functionality. However, classic reversal drugs have two major setbacks: (i) They are not selective, causing side effects in muscarinic receptors that translate into dryness of the mouth and gastrointestinal dysfunctions [95]; and (ii) in spite of the careful dosing of the reversal agent, residual neuromuscular blockage is a common issue in post-operative patients, often leading to respiratory complications [96]. An innovative drug for neuromuscular blockage reversal should thus have a different mechanism of action to avoid these issues.

**Figure 3:**
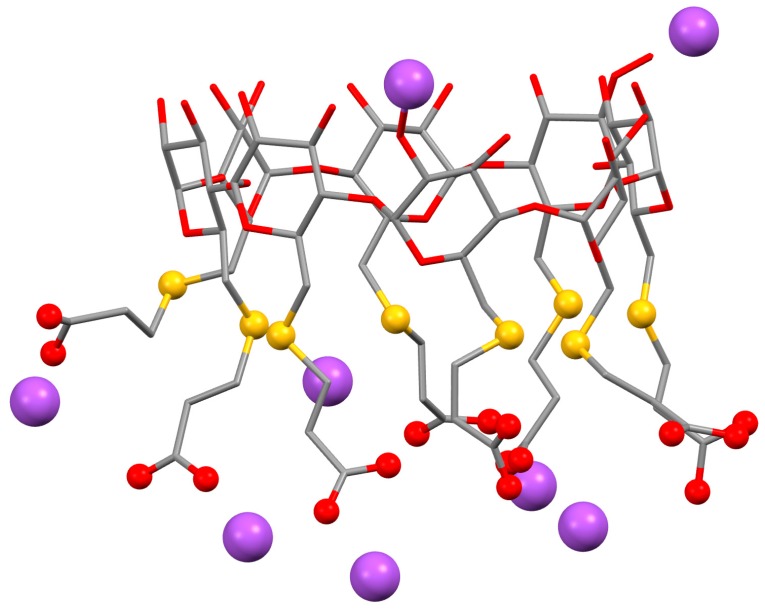
Structures of sugammadex, or octasodium 6A,6B,6C,6D,6E,6F,6G,6H- -octakis-*S*-(2-carboxyethyl)-6A,6B,6C,6D,6E,6F,6G,6-octathio-γ-CD. Hydrogen atoms are omitted for clarity. The sodium cations are highlighted as purple spheres and the functional groups of the substituents are highlighted as red spheres for the oxygens in the carboxyl groups and yellow spheres for the sulphur atoms of the thioether bonds; the remaining structure is represented as sticks, with the red color for oxygen atoms and grey for carbons. Images were redrawn using the software package Mercury from the crystallographic coordinates of the complex, available at the CCDC under the refcode IDIVOG [95].

A simple solution is to use a cyclodextrin that removes the neuromuscular blocking agent from its site of action and renders it inactive by entrapping it in its cavity. For this, rocuronium bromide, one of the most used drugs for surgeries requiring tracheal intubation [97], was chosen as model drug and a γ-CD derivative was designed for maximal inclusion affinity. Of the three native CDs, γ-CD has the best suited inner diameter to include rocuronium bromide, a bulky steroidal guest. The new derivative, called sugammadex, is obtained by perfunctionalization of the primary hydroxyl side of γ-CD with sulphanylpropanoic acid [95], which adds eight linear arms to the base structure and affords a strong increase in cavity depth. Note also that the thiopropanoic arms of sugammadex present themselves in the anionic form (with sodium counterions), which gives this host a quite unique polarity (Figure 3).

Sugammadex forms a very stable complex with rocuronium, being estimated that for every 25 million sugammadex·rocuronium complexes available in an aqueous medium, only one complex dissociates. Sugammadex received approval as a new drug by the EMA in 2008 and by the FDA in 2016 [98]. Sugammadex, administrated intravenously at doses of 2 to 4 mg/kg of body weight, is indicated for the reversal of neuromuscular blockage induced by rocuronium bromide, and also by vecuronium bromide and pancuronium bromide (albeit with lower affinity for the latter case). The time needed by sugammadex to revert rocuronium-induced neuromuscular blockade is much shorter (c.a. 1.5 min) than that for neostigmine (c.a. 9 min) [98,99] and it does not cause residual neuromuscular blockage (unless, of course, an insufficient dose is used) [100]. Clearance from the body also occurs rapidly, within 24 h, and it is done by the kidneys, in a practically unaltered form [101], which can most logically be attributed to the strong polarity and good aqueous solubility of sugammadex. The counter-indications of this drug are also associated with the renal clearance: Sugammadex is not indicated for use in patients with renal failure, in which the sugammadex·rocuronium inclusion complex may not be fully eliminated even after seven days [102].

## 3. Cyclodextrins in Biomedical Technology

### 3.1. Semen Cryopreservation

Preservation of semen through freezing is a practical and cost-effective process of widespread use in the industry of poultry and cattle and also in the preservation of the lineage of horse stallions. The semen is usually diluted in an appropriate medium, which may be egg yolk, a buffer, and/or glycerin. Supplementation of the dilution medium with cyclodextrins is under study. Mammal species seem to require cholesterol along with the cyclodextrins, while in poultry cryopreservation works better when pure cyclodextrins are used.

Cryopreservation of stallion semen usually employs egg yolk for dilution that can be successfully replaced by cholesterol-loaded HPβCD. The HPβCD·cholesterol complex allows cholesterol to be conveyed to the medium in a water-soluble manner and it brings the extra benefit of improving semen viability and mobility after thawing [103]. Cryopreservation of goat sperm with cyclodextrins can also be achieved following a similar strategy [104,105]. Excess amounts of the RAMEB·cholesterol complex are added to the dilution medium of the sperm prior to freezing, with the objective of letting these cells lose less membrane cholesterol during the cryopreservation process. A 30% reduction of membrane cholesterol loss was confirmed by fluorescence microscopy. These spermatozoa can better endure the cold shock, but their fertility rate still did not increase [104]. The RAMEB·cholesterol complex is also useful in the cryopreservation of the sperm of other mammal species, namely Markhoz (Angora) bucks (*Capra hircus*) [106], buffalo bull [107], and boar [108].

The effects of adding pure RAMEB to mammalian sperm are still not well established. RAMEB addition has been reported to cause dose-dependent deleterious effects, meaning that the dose must be carefully pondered. For instance, adding RAMEB to buffalo sperm at doses of 1, 2, 4, and 8 mg/mL brings a dose-dependent increase in sperm capacitation (an important maturation step required for fertilization), albeit at higher doses (4 and 8 mg/mL). RAMEB also causes a loss in sperm motility [109], thus stressing the need for case-to-case dose adjustment. Moreover, in bovine sperm, RAMEB supplementation causes a dose-dependent reduction of viability, with spermatozoa being less viable than the control by c.a. 22% when treated with 1.3 mg/mL (1 mM) RAMEB and by c.a. 40% when treated with 6.5 mg/mL (5 mM) RAMEB [110].

In chicken sperm preservation, adding pure HPβCD [111,112] and RAMEB [111] benefits sperm motility and viability after thawing. Interestingly, adding cholesterol in tandem with the cyclodextrins seems to be detrimental, as chicken sperm supplemented with HPβCD·cholesterol complex exhibits reduced motility and the one added with RAMEB·cholesterol exhibits apoptosis and damage of the acrosomes and of the cell walls of the spermatozoa [111].

### 3.2. Biomimetic Corneal Implants

Corneal blindness affects around 5 million people [113]. The origins are multifactorial, including hyperkeratinisation associated with ophthalmic infections or small eye injuries [114] and corneal dystrophies of genetic origin [113]. The treatment involves replacement of the cornea by transplantation of another human cornea, harvested post-mortem from volunteer organ donors. Besides the limitations associated with the low number of donors, allograft corneal transplantation is prone to immune rejection [115]. An ideal solution would be to produce biomimetic artificial corneas with adequate transparency, mechanical resistance, and biocompatibility.

**Figure 4:**
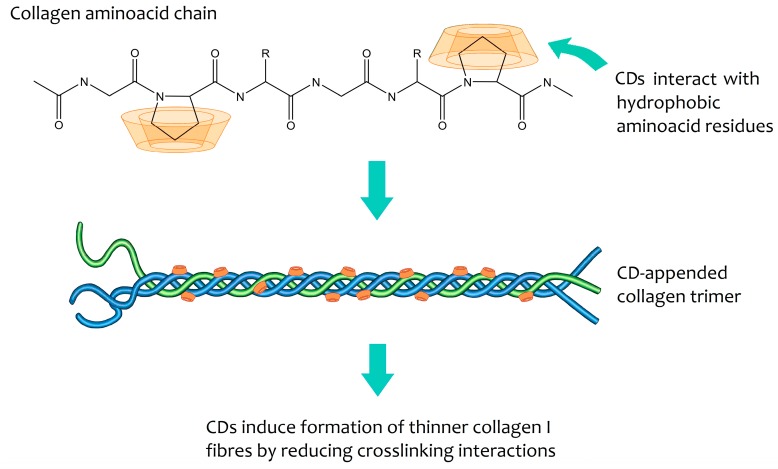
Mode of action proposed for in vitro modulation of collagen growth by cyclodextrins. Although the precise mode of inclusion is not known, CDs are postulated to interact with hydrophobic residues of amino acids present in single chains during their formation. The amino acid chains associate into trimers and these trimers subsequently group into fibrils. The presence of cyclodextrins reduces the crosslinking between the various trimers that form the fibrils, thus leading to the formation of thin fibrils and type I collagen [116].

Cyclodextrins can help bioengineer the growth of collagen in vitro [116], making its structure similar to the one found in the cornea. Collagen fibrils in the cornea have very particular properties, being narrower than the fibrils in other connective tissues to ensure transparency. When cyclodextrins are added to collagen during fibrinogenesis, they interact with hydrophobic amino acid residues of collagen, thereby interrupting the crosslinking process (Figure 4) [116]. Transparent and mechanically robust corneal substitutes can be engineered using native cyclodextrins to modulate the type I collagen self-assembly process during vitrification. Not only is the ultrastructure of these biomimetic corneal substitutes similar to that of the native cornea, as saturation tests using the β-CD/collagen cornea also show that it is resistant enough and supports re-epithelialisation and host tissue integration [115]. These materials have huge potential as biomimetic cornea substitutes, and the technology is already protected by patents [117,118].

### 3.3. Joint Fillers for Arthritis

The use of CDs in tissue engineering applications, such as joint or bone trauma and arthritis, is currently under development. The association of collagen with β-CD, for instance, can help form new bone and cartilage. When incorporated into a model scaffold made of collagen and glycoseaminoglycan, β-CD was shown to influence the metabolic activity and proliferation of mesenchymal stem cells, driving them towards osteo-chondral differentiation. This happens because β-CD forms inclusion complexes with two growth factors, TGF-β1 and BMP-2, and then releases them in a slow, sustained fashion [119]. A joint filler comprising an association of β-CD or HPβCD with hyaluronate and chondroitin is patented for use in the treatment or prevention of cartilage degeneration and arthrosis or arthritis diseases, or as a filling agent for soft tissues and mucosae [120].

## 4. Conclusions

The present review described how cyclodextrins are steadily making their way into medicinal applications, both as APIs and in biomedical engineering. Approved medicinal cyclodextrins include sugammadex and HPβCD. Sugammadex is an anionic γ-CD derivative designed to revert muscular blockage caused by the rocuronium bromide. Sugammadex includes rocuronuim bromide with high specificity and a very strong affinity; this entrapment into the sugammadex cavity ensures the muscular blockage effect is quickly terminated. HPβCD is approved as an orphan drug for the treatment of a rare kidney disease, focal segmental glomerulosis, and for compassionate use in the treatment of a degenerative brain disease, Niemann pick disease type C. In these two diseases, the pathophysiology involves the accumulation of cholesterol in the cells or the living tissue, and the action of HPβCD is thought to be related to its ability to sequester cholesterol and remove it from the damaged sites.

Cholesterol inclusion by HPβCD and other CD derivatives opens way for various clinical uses, namely the control of viral infections and of parasitic infections, such as malaria, leishmaniasis, or criptosporidiasis. The use of CDs for the removal of cholesterol and other lipids that build up in the arteries and atherosclerosis plaques is also under study, with interesting preliminary results. Further studies are needed confirm the utility of CDs in the management of cardiovascular disease. Atherosclerosis develops by complex processes and for this reason the activity of CDs is not expected to be limited to cholesterol and lipids. It is necessary to investigate the possible interaction of CDs with inflammation signaling molecules and with immune cells occurring at the site of the atherosclerosis plaque, such as macrophages.

Cyclodextrins also interact with collagen, which makes them promising modulators for medicinal biomaterials. Under development are biomimetic corneas prepared by growing collagen in a CD-containing medium as well as filling materials for arthritis-damaged joints made of CDs in association with collagen and glycosaminoglycan.

The examples presented in this review are the early stage of a new era of medicinal cyclodextrins. The excellent biocompatibility of CDs, their wide range of possible interactions with biomolecules, and the flexibility of functionalization to obtain derivatives that will interact specifically with a selected biomolecular target give them a vast medicinal potential, and many more applications are expected to arise in the near future.

## Abbreviations

API	Active pharmaceutical ingredient
BALB/c	Bagg albino mouse (inbred research mouse strain)
BMP-2	Bone morphogenetic protein 2 (a subclass of TGF)
CCDC	Cambridge Crystallographic Data Centre
CD	Cyclodextrin
CNS	Central nervous system
DEN	Dengue virus
DIMEB	Heptakis-2,6-di-*O*-methyl-β-cyclodextrin
EMA	European Medicines Agency
FAO	Food and Agriculture Organization of the United Nations
FDA	Food and Drug Administration
FSGS	Focal segmental glomerulosclerosis
GRAS	Generally regarded as safe
H1N1	Influenza virus type A subtype with hemmagluttinin 1 and neuraminidase 1
HCV	Hepatitis C virus
HMPV	Human metapneumovirus
HPβCD	Hydroxypropylated-β-cyclodextrin (in random positions)
HPIV3	Human parainfluenza virus type 3
HSV-1	Herpes simplex virus type 1
IBV	Infectious bronchitis virus
IL-10	Interleukin 10
JECFA	Joint FAO/WHO Expert Committee on Food Additives
JEV	Japanese encephalitis virus
LAMP-1	Lysosomal-associated membrane protein 1
MDCK	Madin-Darby Canis familiaris kidney epithelial cell line
NDV	Newcastle disease virus
NPD	Niemann-Pick disease
NS1	Non-structural protein 1
RAMEB	Randomly methylated β-cyclodextrin
SIV	Simian immunodeficiency virus
TRIMEB	Heptakis-2,3,6-tris-*O*-methyl-β-cyclodextrin
TNF-α	Tumour necrosis factor alpha
U937	Homo sapiens pleural myelomonocyte cell line
VZV	Varicela-zooster virus (chickenpox virus)
WHO	World Health Organisation
TGF-β1	Transforming growth factor beta 1 (cytokine family)
